# Supplementary material for: ORP2 couples LDL‐cholesterol transport to FAK activation by endosomal cholesterol/PI(4,5)P2 exchange
Source: EMBO J. 2021 Jun 14;40(14):e106871. doi: 10.15252/embj.2020106871 (PMC8281050; doi:10.15252/embj.2020106871)
Supplement: Supplementary file 1 — Appendix [file EMBJ-40-e106871-s008.pdf]

Appendix

Table of contents

Appendix Figure S1. Antibody validation 2

Appendix Figure S2. Edited genome sequence in degron-GFP-ORP2 cells 3

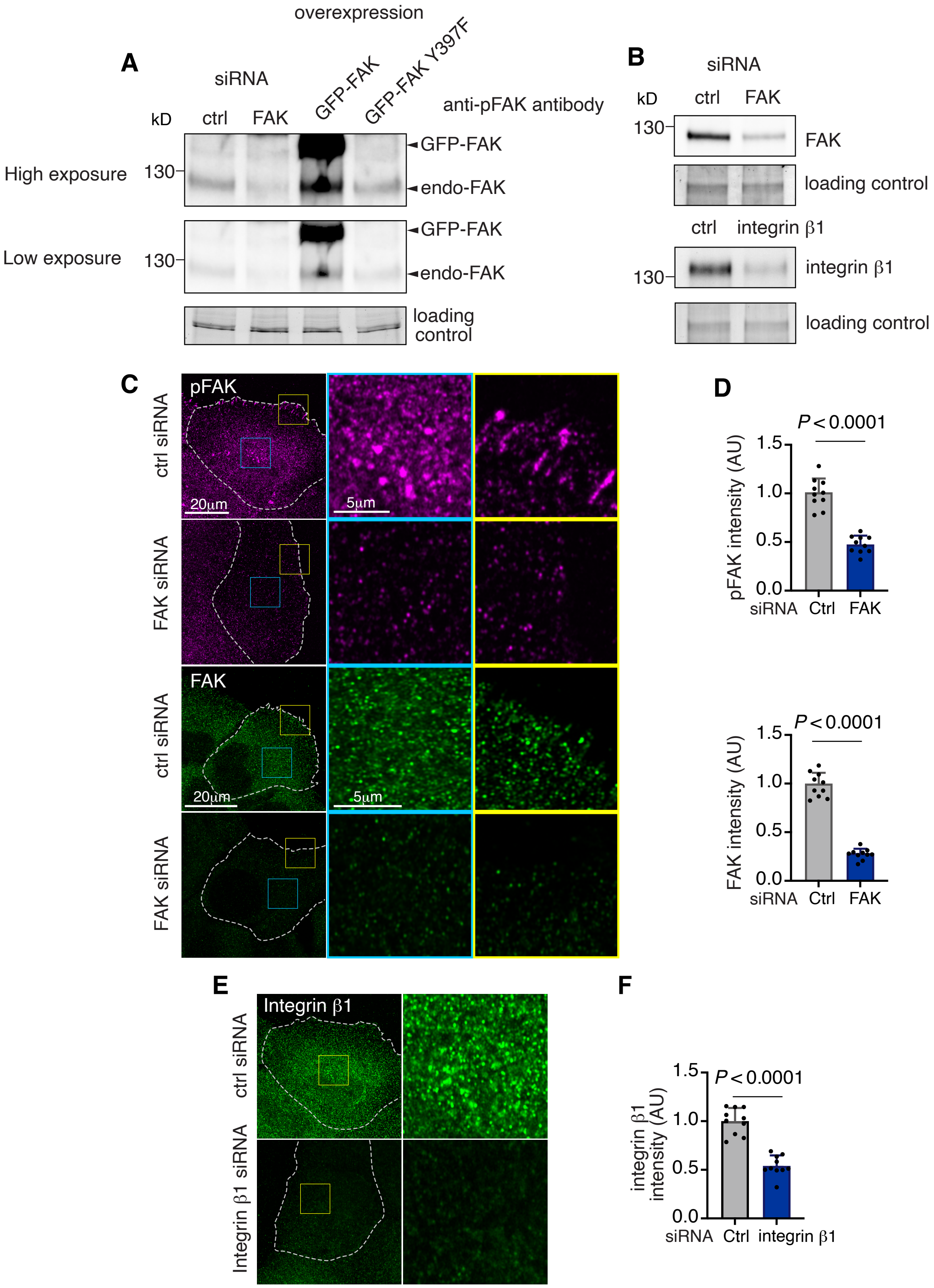

Appendix Figure S1. Antibody validation.

A, Cells were treated with control or FAK siRNAs for 3 days, or transfected with GFP-FAK or GFP-FAK Y397F for 1 day and cell lysates immunoblotted with anti-pFAK antibodies (BD Transduction Laboratory #611807, clone 18).
B, Cells were treated with control, FAK or integrin β1 siRNAs for 3 days and immunoblotted with anti-FAK (Sigma 05-537, Clone 4.47) or anti-integrin β1 (Sigma MAB2252, clone N29) antibodies.
C, Cells were treated with control or FAK siRNAs for 3 days, fixed and immunostained with anti-pFAK (Thermo Fisher #44-624G) or anti-FAK antibodies (Sigma 05-537, Clone 4.47).
D, Quantification of signal intensities in C. Mean ± SD, n = 10 cells. Student's t-test.
E, Cells were treated with control or integrin β1 siRNAs for 3 days, fixed and immunostained with anti-integrin β1 antibodies (Santa Cruz sc18887, K20).
F, Quantification of D. Mean ± SD, n = 10 cells. Student's t-test.

Forward primer: TTCTGGAAGCTAAGTATGC
Reverse primer: CACACACTGCTTTACTGAG

ORP2 genome
Start codon
miniIAA7
EGFP
Linker

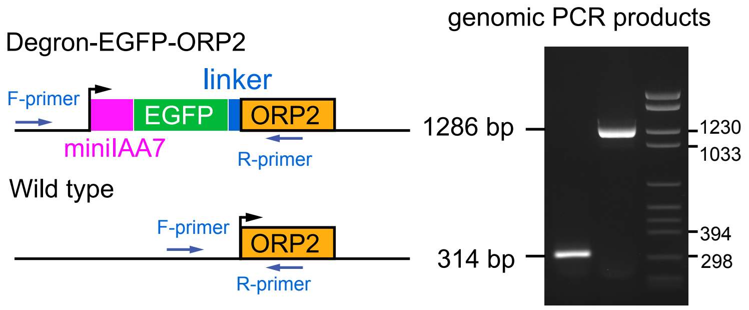

Genome sequence of Degron-EGFP-ORP2 cells:

AAGTTTGTAAAATTCCTTACACTGTAGATGTGGATCAGATACGATGATTCAGTAGAAGAG-
CACATGTCAGGGGCAGTGGAGGCTGGCTGCTGAAGGATGGAGGGCTTCTCTGAGACCGTGGA
CCTGATGCTGAACCTGCAGTCCAATAAGGAGGGCTCTGTGGATCTGAAGAACGTGAGCGCCGTG
CCTAAGGAGAAGACCACACTGAAGGACCCATCCAAGCCCCCTGCCAAGGCACAGGTGGTGGGA
TGGCCACCCGTGCGGAACTACAGAAAGAATATGATGACCCAGCAGAAGACAAGCTCCTCCGGA
GTGAGCAAGGGCGAGGAGCTGTTCACCGGGGTGGTGCCCATCCTGGTCGAGCTGGACGGCGA
CGTAAACGGCCACAAGTTCAGCGTGTCCGGCGAGGGCGAGGGCGATGCCACCTACGGCAAGCT
GACCCTGAAGTTCATCTGCACCACCGGCAAGCTGCCCGTGCCCTGGCCCACCCTCGTGACCACC
CTGACCTACGGCGTGCAGTGCTTCAGCCGCTACCCCGACCACATGAAGCAGCACGACTTCTTCA
AGTCCGCCATGCCCGAAGGCTACGTCCAGGAGCGCACCATCTTCTTCAAGGACGACGGCAACTA
CAAGACCCGCGCCGAGGTGAAGTTCGAGGGCGACACCCTGGTGAACCGCATCGAGCTGAAGG
GCATCGACTTCAAGGAGGACGGCAACATCCTGGGGCACAAGCTGGAGTACAACTACAACAGCC
ACAACGTCTATATCATGGCCGACAAGCAGAAGAACGGCATCAAGGTGAACTTCAAGATCCGCCA
CAACATCGAGGACGGCAGCGTGCAGCTCGCCGACCACTACCAGCAGAACACCCCCATCGGCGA
CGGCCCCGTGCTGCTGCCCGACAACCACTACCTGAGCACCCAGTCCAAGCTGAGCAAAGACCCC
AACGAGAAGCGCGATCACATGGTCCTGCTGGAGTTCGTGACCGCCGCCGGGATCACTCTCGGCA
TGGACGAGCTGTACAAGACCGGAGGCGGAGGGAGCGGGGGAGGCGGATCTGGCGGAGGCG
GATCCAACGGAGAGGAAGAATTCTTTGATGCCGTCACAGGTGAGTCAAAAGAGAACCAACTGG
GGACGTACTGGAAGGGTGAACGTCCCTGGATT

Appendix Figure S2. Edited genome sequence in degron-GFP-ORP2 cells.
Genomic DNA was extracted from degron-GFP-ORP2 cells, amplified with the indicated primers, and DNA sequencing was performed using the same primers. Genomic PCR amplification products are shown. The sequences corresponding to ORP2 genome, start codon, miniIAA7, EGFP and linker are color coded.
