# Supplementary material for: ORP2 couples LDL‐cholesterol transport to FAK activation by endosomal cholesterol/PI(4,5)P2 exchange
Source: EMBO J. 2021 Jun 14;40(14):e106871. doi: 10.15252/embj.2020106871 (PMC8281050; doi:10.15252/embj.2020106871)
Supplement: Supplementary file 2 — Expanded View Figures PDF [file EMBJ-40-e106871-s001.pdf]

Expanded View Figures

Figure EV1. D4H cholesterol binding, and FAK and cholesterol dependence of FA dynamics.

A sfGFP-D4H binding to GUVs. The lipid composition was POPC: rhodamine-DHPE (99:1, mol/mol) (left panel) and POPC:cholesterol:rhodamine-DHPE (69:30:1, mol/mol) (right panel). Images were acquired by widefield epifluorescence microscopy.

B Cells stably expressing mCherry-talin were generated and the FA dynamics was imaged using TIRF microscopy and quantified. Assembly rate: n = 242 FAs (−LDL); 454 (+LDL); 280 (+LDL+PF-228), disassembly rate: n = 325 (−LDL); 654 (+LDL); 366 (+LDL+PF-228), from 4 independent experiments.

C Cells were transiently transfected with GFP-FAK or GFP-FAK Y397F, and FA dynamics was imaged using TIRF microscopy and quantified. Assembly rate: n = 321 FAs (−LDL, FAK); 244 (+LDL, FAK); 149 (+LDL, Y397F), disassembly rate: n = 363 (−LDL, FAK); 341 (+LDL, FAK); 184 (+LDL, Y397F), from 4 independent experiments.

D Cells were treated with 5 mM MβCD for 30 min and FA assembly and disassembly rates were quantified. Assembly rate: n = 153 FAs (control); 66 (MβCD), disassembly rate: n = 190 (control); 88 (MβCD), from 4 independent experiments.

E Quantification of LDL-induced FA dynamics upon LDL loading ± 2 μg/ml U18666A for the indicated times. Assembly rate: n = 242 FAs (−LDL); 537 (LDL 0–1 h); 454 (LDL 1–2 h); 1057 (LDL 2–3 h); 846 (LDL 3–4 h); 523 (LDL 1–2 h + U18666A), disassembly rate: n = 325 (−LDL); 777 (LDL 0–1 h); 654 (LDL 1–2 h); 1382 (LDL 2–3 h); 1313 (LDL 3–4 h); 550 (LDL 1–2 h + U18666A), from 4 independent experiments. Non-parametric Kruskal–Wallis analysis of variance with Bonferroni error correction. The Tukey box plots represent the median and 25th and 75th percentiles (interquartile range). The whiskers show the highest and lowest observations.

F Cells were loaded with 200 μM cholesterol/ MβCD complex for the indicated times and pFAK and FAK were detected by immunoblotting.

Source data are available online for this figure.

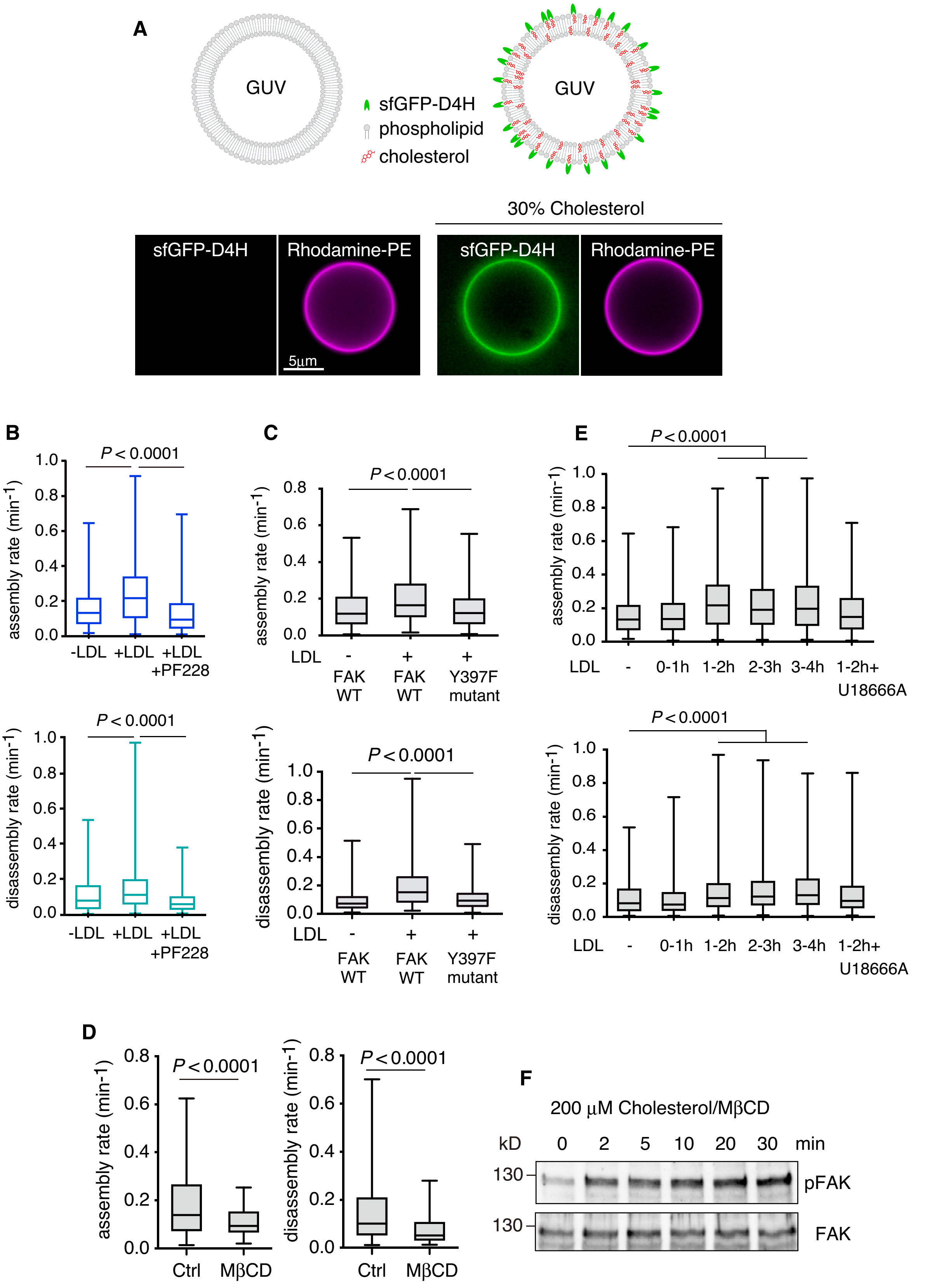

Figure EV1.

 © 2021 The Authors

Figure EV2. LDL-derived BODIPY-cholesterol delivery to integrin endosomes.

A Confocal images of live A431 cells labeled overnight with dextran (magenta) followed by 2 h pulse loading with BODIPY-cholesterol (green) ester-labeled LDL and imaged immediately before chase. Dashed lines indicate cell outlines.

B Confocal micrographs of BODIPY-cholesterol (green) and integrin β1 (magenta) imaged at the indicated time points of LDL pulse-chase assay presented in Fig 2A and quantified in Fig 2B. Dashed lines indicate cell outlines.

C Schematic of live-cell BODIPY-cholesteryl linoleate LDL pulse chase with AF647-labeled transferrin.

D Confocal imaging of BODIPY-cholesterol (green) and transferrin (magenta) at the indicated time points of the assay presented in Fig EV2C. Arrowheads indicate colocalization. Dashed lines indicate cell outlines.

E Quantification of BODIPY-cholesterol fluorescence in transferrin organelles, related to EV2D. Mean ± SD, $n = 10$ cells from 2 independent experiments. Student's *t*-test.

F Time series stills of an example of BODIPY-cholesterol (green) organelles contacting an integrin β1 (magenta)-positive organelle and sharing the cargo during LDL pulse-chase assay. Confocal stills are from Movie EV1. A-C mark individual organelles; integrin β1-positive organelle (A), BODIPY-cholesterol positive organelle contacting (B) or not contacting (C) integrin β1-positive organelle A.

G Quantification of BODIPY-cholesterol transfer in contacting vs. non-contacting integrin β1-organelles shown in EV2F, Mean ± SEM, $n = 10$ organelles from 3 independent experiments. Student's *t*-test.

© 2021 The Authors

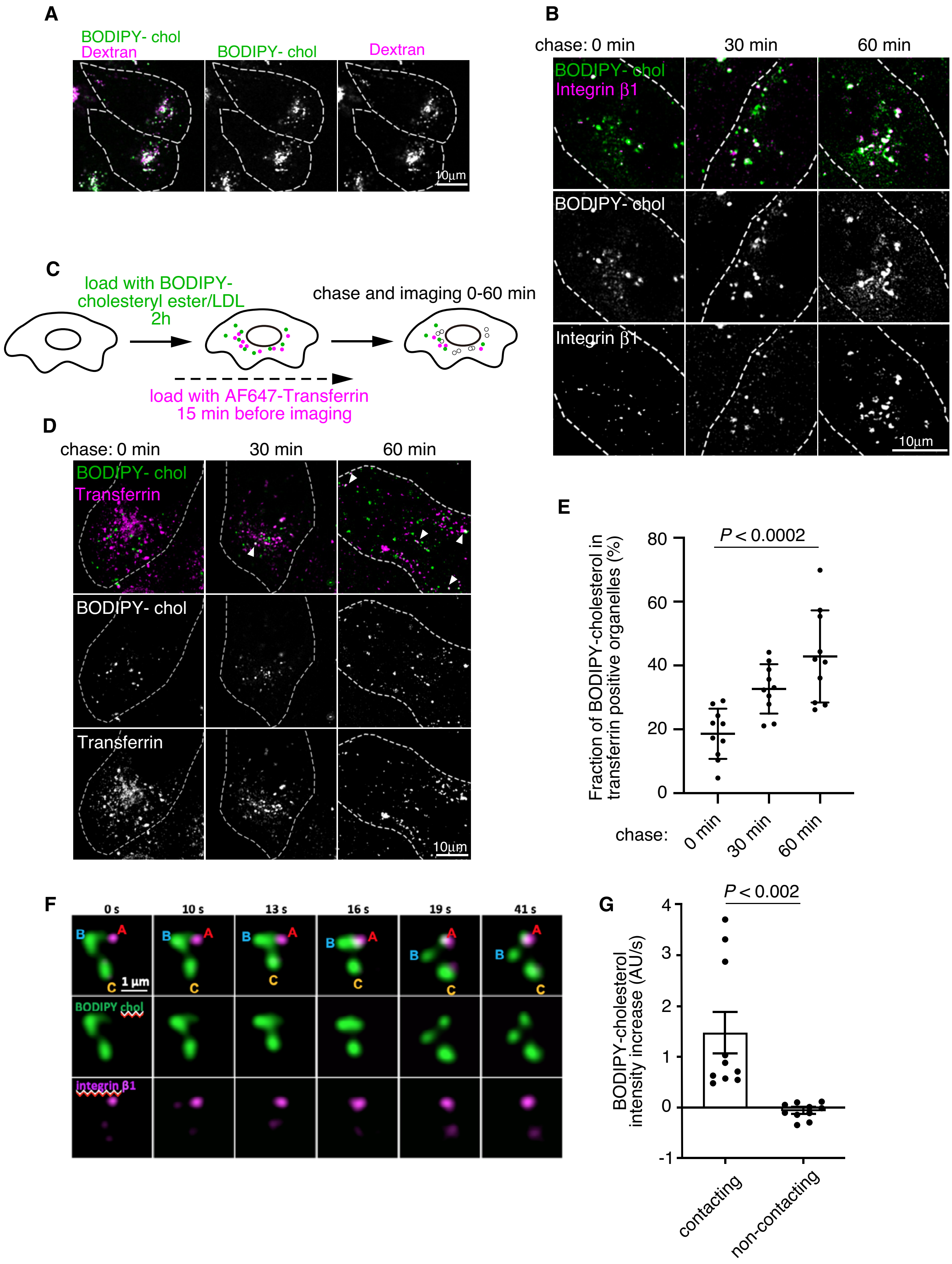

Figure EV2.

© 2021 The Authors

Figure EV3. FAK inhibition abrogates LDL-cholesterol delivery to the PM.

A Cells were seeded on fibronectin-coated coverslips, incubated in complete medium (CM) or 5% LPDS overnight, treated with 10 μM PF-228 for 30 min as indicated, followed by incubation with 10 μg/ml DiI-LDL in serum-free medium for 1 h + 10 μM PF-228 as indicated, fixed with 4% PFA for 15 min, and mounted. Images were acquired by widefield epifluorescence microscopy, and DiI-LDL intensity per cell area was quantified. Mean ± SD, n = 23–57 cells from 2 independent experiments.

B After 1 day in 5% LPDS, cells were loaded with 50 μg/ml LDL ± 10 μM PF-228 for the indicated times, PM cholesterol was labeled with D4H as in Fig 1A and imaged by widefield epifluorescence microscopy. Dashed lines indicate cell area.

C Quantification of (B). Mean ± SD, n = 15 fields of cells from 2 independent experiments. Student's t-test.

D, E Exemplary images corresponding to quantifications in Fig 3F and G. Dashed lines indicate cell outlines.

F, G After 1-day 5% LPDS incubation, cells were transfected with iRFP-PLC PH for overnight. Cells were loaded with LDL ± 10 μM PF-228 for 4 h and imaged live by confocal microscopy (F). Line scan analysis of the regions indicated by white lines in the images. Intensity ratio of iRFP-PLC PH (plasma membrane/cytosol) was quantified (G). Mean ± SD, n = 30–43 cells pooled from 2 independent experiments. Student's t-test.

© 2021 The Authors

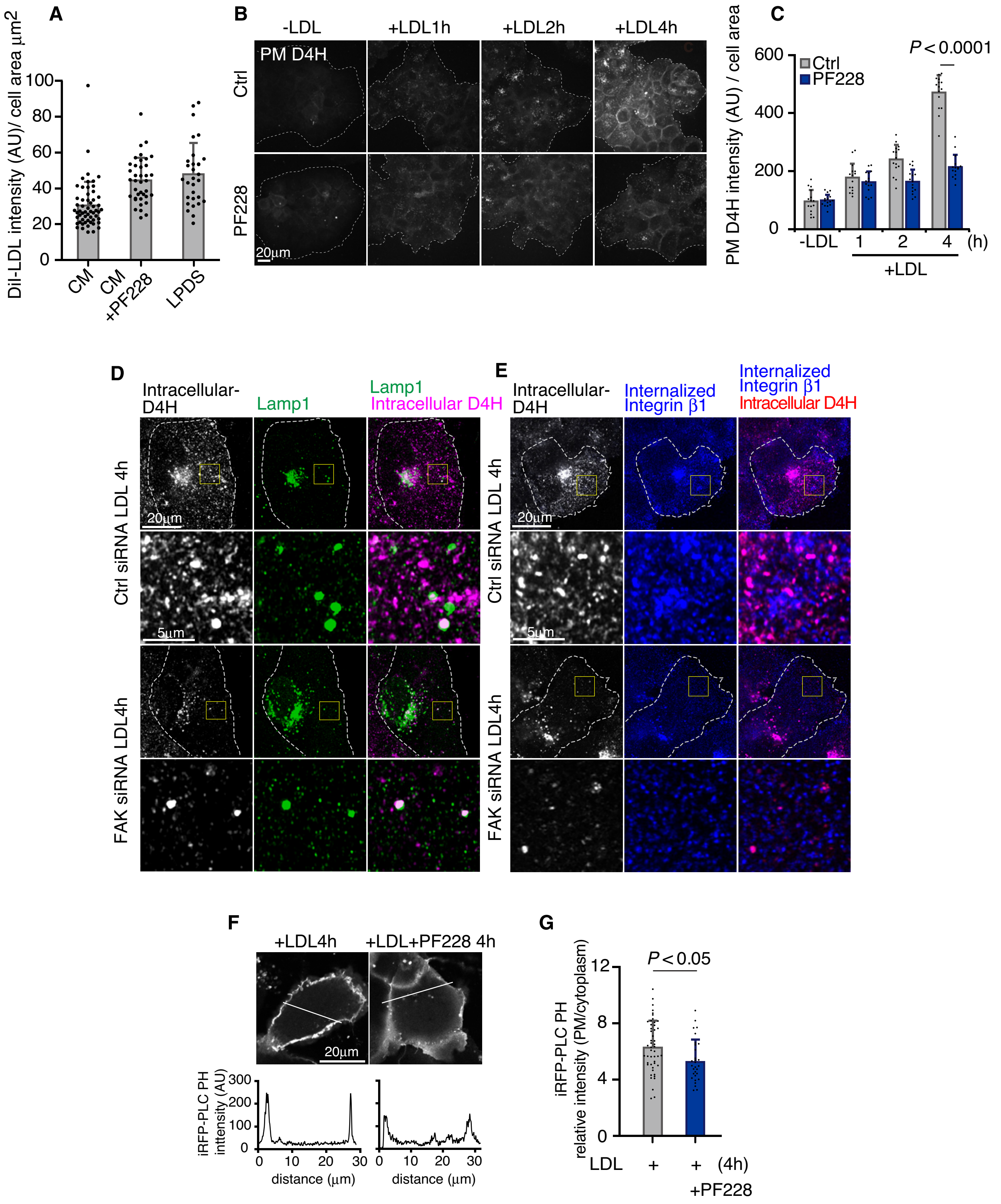

Figure EV3.

 © 2021 The Authors

Figure EV4. ORP2 depletion abrogates LDL-cholesterol delivery to the PM and LDL-induced cell adhesion.

A Cells were transfected with the indicated siRNA for 2 days, incubated in 5% LPDS for 1 day, loaded with 50 µg/ml LDL for the indicated times, fixed, and stained for PM D4H. Images were acquired by widefield epifluorescence microscopy. CM, complete medium.

B Quantification of PM D4H staining in (A). Mean ± SD, n = 20 fields of cells as in (A), from 2 independent experiments. Student's t-test.

C Immunoblots of endogenous non-tagged ORP2 (WT cells) and endo-GFP-ORP2 (degron-ORP2 control and degron-ORP2 cells) treated with IAA for the indicated times. The band at ~ 90 kD is unspecific.

D Degron ORP2 cells were incubated without (−IAA: ORP2 present) or with IAA (+IAA; ORP2 depleted) for 1 or 6 h, then with 10 µg/ml DiI-LDL in serum-free medium for 1 h, fixed with 4% PFA for 15 min and mounted. Images were acquired by widefield epifluorescence microscopy. Mean ± SD, n = 10 fields of cells from 2 independent experiments.

E, F Degron ORP2 cells were incubated ± IAA for 3 days, transfected with iRFP-PLC PH for overnight, and imaged live by confocal microscopy. Line scan analysis of the regions indicated by white lines in the image. The fluorescence intensity ratio between plasma membrane and cytosol was quantified. Mean ± SD, n = 28–30 cells from 2 independent experiments. Student's t-test.

G Degron ORP2 cells were transfected with iRFP-PLC PH in 5% LPDS for 1 day, loaded with LDL + IAA for 1 or 4 h and iRFP-PLC PH intensity quantified as in EV4F. Mean ± SD, n = 28–30 cells from 2 independent experiments. Student's t-test.

H Degron ORP2 cells were treated ± IAA for 4 h or with 5 mM MβCD for 1 h and cellular transferrin uptake was quantified. MβCD causes extensive cholesterol depletion and was used as a positive control. Mean ± SD, n = 4 independent experiments. One-way ANOVA with Tukey's post hoc test.

I–K Degron ORP2 cells were treated as indicated in (I), trypsinized, replated on fibronectin-coated coverslips in ± LDL ± IAA for 15, 30, or 60 min. Cells were fixed in 4% PFA for 15 min and stained with pFAK. Representative confocal images of pFAK immunostaining after replating (J). Quantification of cell area after replating (K). Mean ± SD, n = 30 cells.

Source data are available online for this figure.

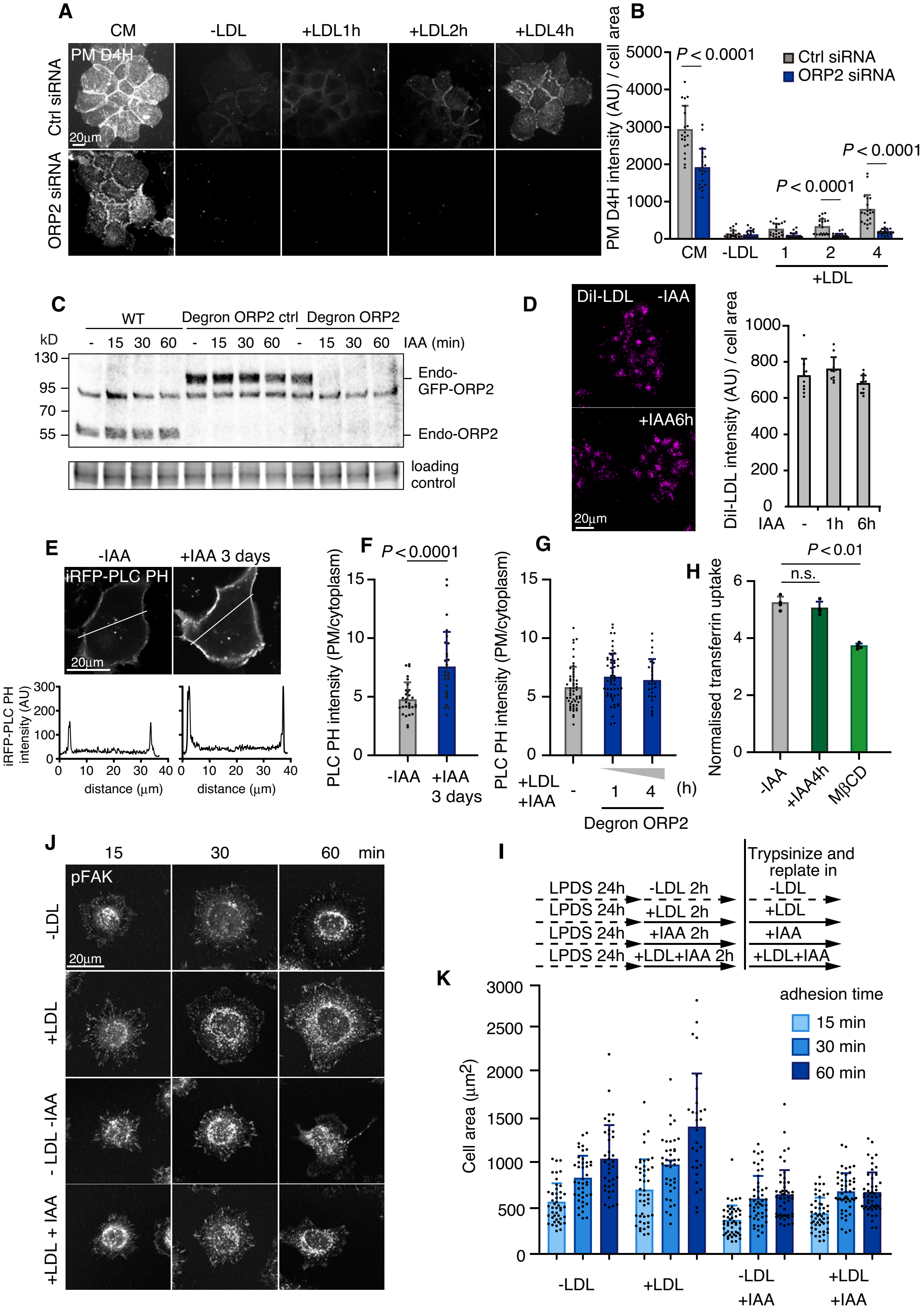

Figure EV4.

 © 2021 The Authors

Figure EV5. Effects of ORP2 depletion or FAK inhibition on endomembranes and FAK phosphorylation, and FAK FERM domain binding to PI(4,5)P_2-containing membranes.

A Schematic of live-cell dextran and transferrin pulse-chase in degron-ORP2 cells.

B Exemplary confocal images from movies of +/− IAA-treated degron-ORP2 cells labeled with AF647-transferrin (magenta) and fluorescein dextran (green) as in Fig EV5A. Trajectories of manually tracked double-positive organelles at 30 min chase time point of the corresponding time series shown in Movie EV2 (-IAA; ORP2 present) and Movie EV3 (+IAA; ORP2 depleted).

C Duration of contacts between transferrin and dextran organelles. Mean ± SEM, n = 28 contacts from 6 cells (+IAA); 36 contacts from 5 cells (-IAA). Student's t-test. Number of contacts between dextran and transferrin organelles. Mean ± SEM. n = 42 (-IAA); 39 (+IAA) dextran organelles. Student's t-test.

D Control and degron-ORP2 cells were co-plated. After 1 day in 5% LPDS, cells were loaded with 50 μg/ml LDL and IAA for the indicated times, stained with PI(4,5)P_2 antibody, and imaged by confocal microscopy. Asterisk indicates ORP2-depleted cell.

E Quantification of EV5D. Mean ± SD, n = 13–23 cells from 2 independent experiments. Student's t-test.

F, G TIRF imaging of NPC1-GFP distribution in live cells with or without 10 μM PF-228 for 4 h, and quantification of NPC1 vesicles within 10 μm distance from the cell edge (dotted lines). Transiently transfected mCherry-FAK was used to mark the cell edge. Mean ± SD, n = 6 cells. Student's t-test.

H Representative epifluorescent images of NPC1-mCherry organelles in GFP-ORP2 or GFP-ORP2-mHHK overexpressing cells quantified in Fig 6E. Arrowheads indicate tubular NPC1 organelles.

I After 1 day in 5% LPDS, control and degron-ORP2 cells were loaded with 50 μg/ml LDL and IAA for the indicated times, lysed and subjected to immunoblotting with pFAK antibody. Mean ± SD, n = 3 independent experiments. Paired Student's t-test.

J Control and degron-ORP2 cells were co-plated and incubated without (−LDL) or with LDL (+LDL) and IAA (+IAA; ORP2 depletion) for 1 h. Representative confocal images showing increased pFAK intensity at perinuclear endomembranes upon LDL loading in control cells but not in the ORP2-depleted degron cell (asterisks). Red inset shows endomembrane endo-GFP-ORP2 and pFAK signals in the control cell, and blue inset shows corresponding signals in the ORP2-depleted cell. Orange arrowheads indicate mature FAs.

K Representative confocal images of GFP-ORP2-mHHK or GFP-ORP2-ΔELSK-transfected cells quantified in Fig 7B. Dashed lines indicate cell outlines. Asterisks indicate cells depleted of endogenous ORP2.

L AF488-FERM binding to liposomes by liposome-co-sedimentation. Lipid composition and concentration as well as protein concentration were the same as in Fig 7E, in 0, 5, and 10% PI(4,5)P_2-containing liposomes. S; supernatant, P; pellet. Numbers under the blots indicate the fraction of FAK FERM bound to liposomes (P) of total FAK FERM (S + P).

Source data are available online for this figure.

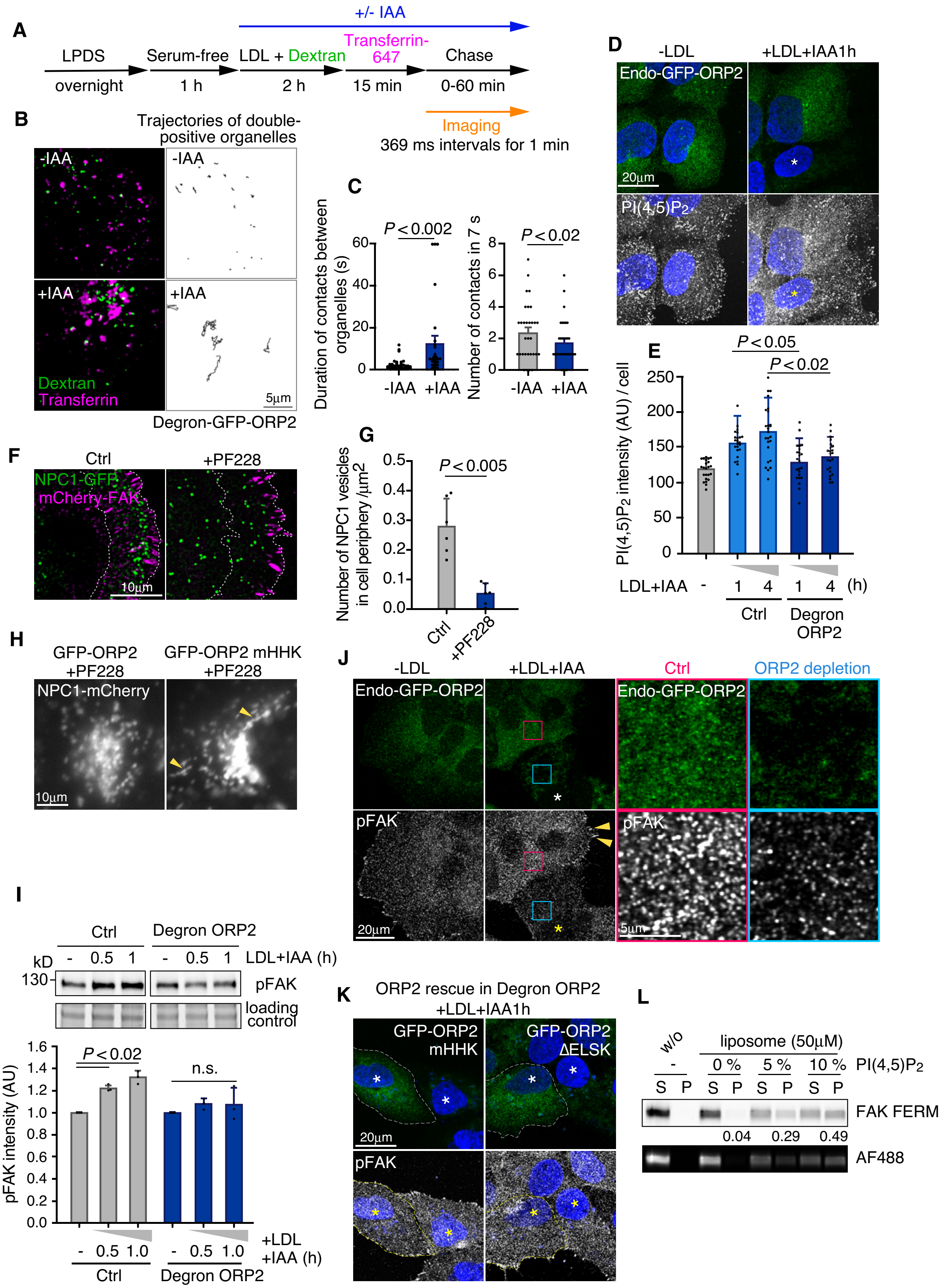

Figure EV5.

 © 2021 The Authors
