# Supplementary material for: ORP2 couples LDL‐cholesterol transport to FAK activation by endosomal cholesterol/PI(4,5)P2 exchange
Source: EMBO J. 2021 Jun 14;40(14):e106871. doi: 10.15252/embj.2020106871 (PMC8281050; doi:10.15252/embj.2020106871)
Supplement: Supplementary file 3 — Movie EV1 [file EMBJ-40-e106871-s003.zip › EMBOJ-2020-106871R3_MovieEV1.docx]

**MovieEV1**

Contacts between BODIPY-cholesterol (green) and integrin β1 (magenta) containing organelles involved in sharing the fluorescent lipid cargo. At 1 h chase, single focal planes of A431 cells were imaged for 1 min with 145 ms frame rate.
