# Supplementary material for: ORP2 couples LDL‐cholesterol transport to FAK activation by endosomal cholesterol/PI(4,5)P2 exchange
Source: EMBO J. 2021 Jun 14;40(14):e106871. doi: 10.15252/embj.2020106871 (PMC8281050; doi:10.15252/embj.2020106871)
Supplement: Supplementary file 4 — Movie EV2 [file EMBJ-40-e106871-s002.zip › EMBOJ-2020-106871R3_MovieEV2.docx]

**MovieEV2**

Dextran (green) and transferrin (magenta) pulse-chase assay in degron ORP2 cells without IAA-treatment. After chasing the cells for 15 min, time-lapse series were captured with 369 ms frame rate for 1 min.
