# Supplementary material for: ORP2 couples LDL‐cholesterol transport to FAK activation by endosomal cholesterol/PI(4,5)P2 exchange
Source: EMBO J. 2021 Jun 14;40(14):e106871. doi: 10.15252/embj.2020106871 (PMC8281050; doi:10.15252/embj.2020106871)
Supplement: Supplementary file 5 — Movie EV3 [file EMBJ-40-e106871-s006.zip › EMBOJ-2020-106871R3_MovieEV3.docx]

**MovieEV3**

Dextran (green) and transferrin (magenta) pulse-chase assay in degron ORP2 cells with IAA-treatment shows fewer and prolonged contacts between dextran-positive late endosomes and transferrin containing recycling organelles. Image acquisition as in Movie EV2.
