# Supplementary material for: ORP2 couples LDL‐cholesterol transport to FAK activation by endosomal cholesterol/PI(4,5)P2 exchange
Source: EMBO J. 2021 Jun 14;40(14):e106871. doi: 10.15252/embj.2020106871 (PMC8281050; doi:10.15252/embj.2020106871)
Supplement: Supplementary file 6 — Movie EV4 [file EMBJ-40-e106871-s005.zip › EMBOJ-2020-106871R3_MovieEV4.docx]

**MovieEV4**

Cells stably expressing NPC1-mCherry were imaged with widefield epifluorescence microscopy, 1 min recording with 1 s frame rate.
