# Supplementary material for: ORP2 couples LDL‐cholesterol transport to FAK activation by endosomal cholesterol/PI(4,5)P2 exchange
Source: EMBO J. 2021 Jun 14;40(14):e106871. doi: 10.15252/embj.2020106871 (PMC8281050; doi:10.15252/embj.2020106871)
Supplement: Supplementary file 7 — Movie EV5 [file EMBJ-40-e106871-s007.zip › EMBOJ-2020-106871R3_MovieEV5.docx]

**MovieEV5**

Cells stably expressing NPC1-mCherry were treated with 10 μM PF228 for 4 h and imaged with widefield epifluorescence microscopy, 1 min recording with 1 s frame rate.
