# Supplementary figures and images for: ORP2 couples LDL‐cholesterol transport to FAK activation by endosomal cholesterol/PI(4,5)P2 exchange
Source: EMBO J. 2021 Jun 14;40(14):e106871. doi: 10.15252/embj.2020106871 (PMC8281050; doi:10.15252/embj.2020106871)

Fig 1F

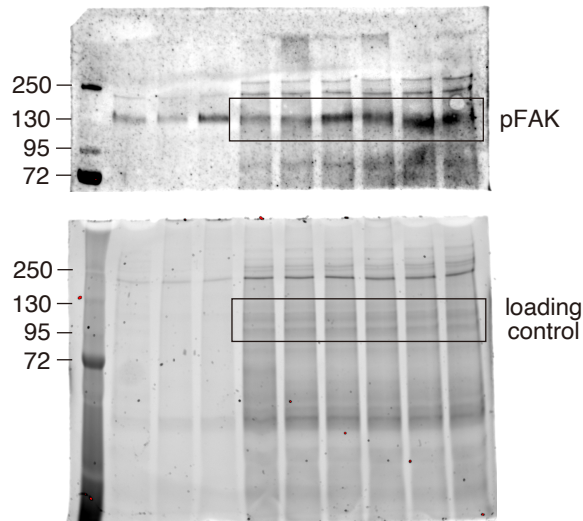

Fig 7C

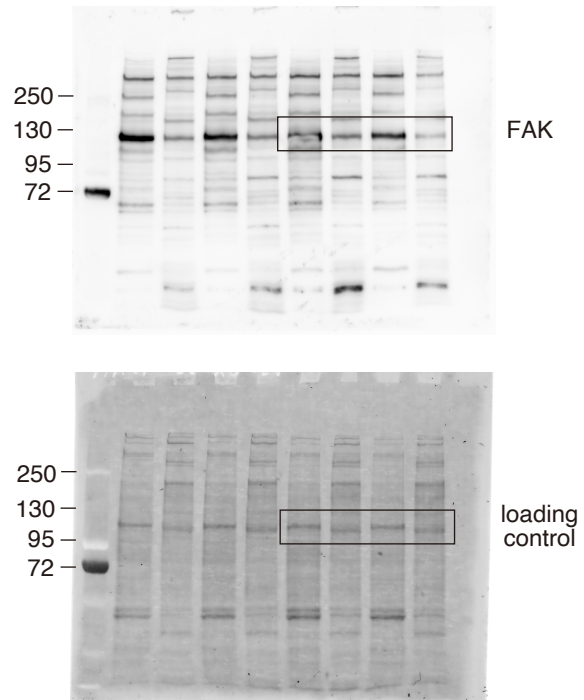

Fig 7E top

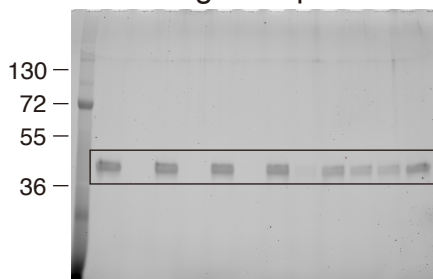

Fig 7E middle

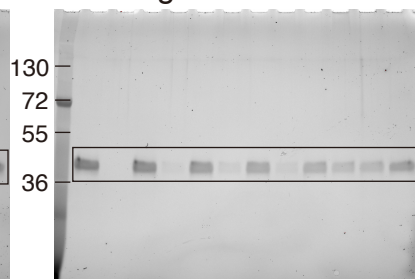

Fig 7E bottom

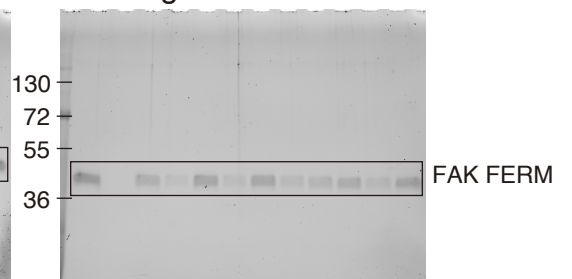

Fig EV1F

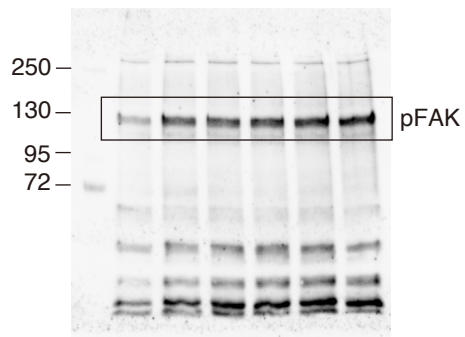

Fig EV4C

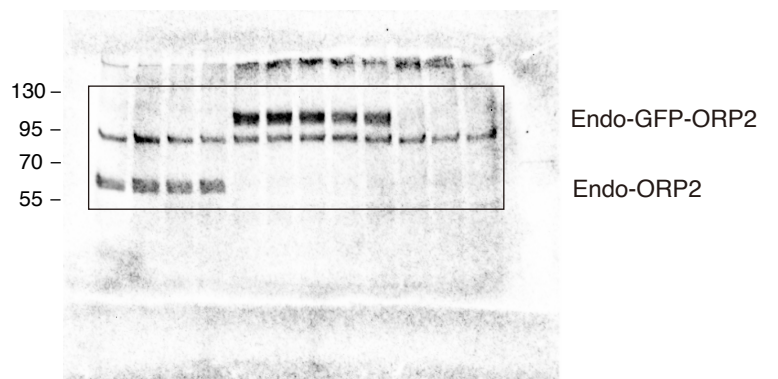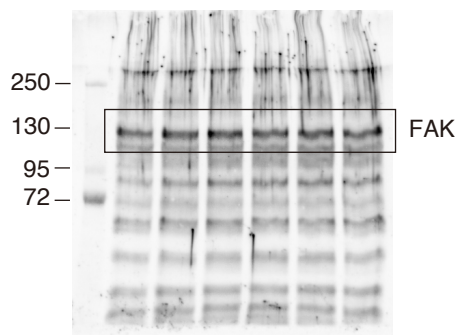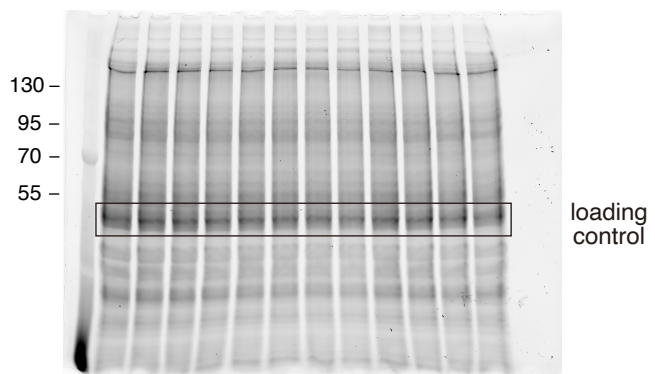

Supplement: Supplementary file 9 — Source Data for Figure [file EMBJ-40-e106871-s004.pdf]

Fig EV5I

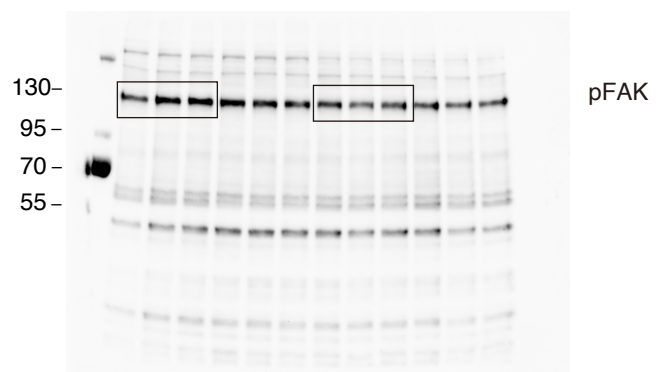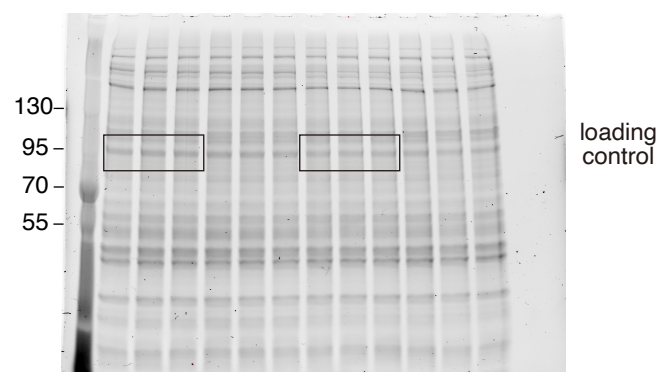

Fig EV5L

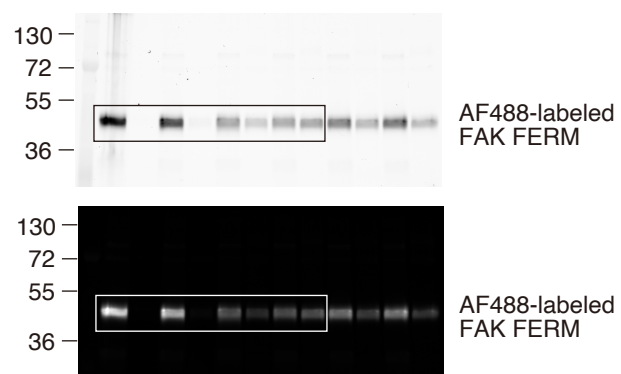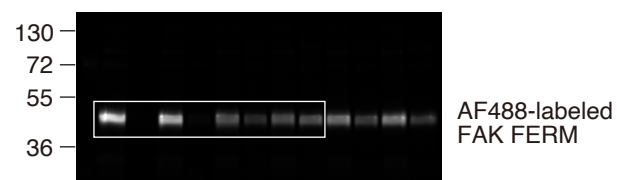

Supplement: Supplementary file 10 — Source Data for Expanded View/Appendix [file EMBJ-40-e106871-s010.zip › EMBOJ-2020-106871R2-Figure_Source_Data2-sd.pdf]

Appendix Figure 1A

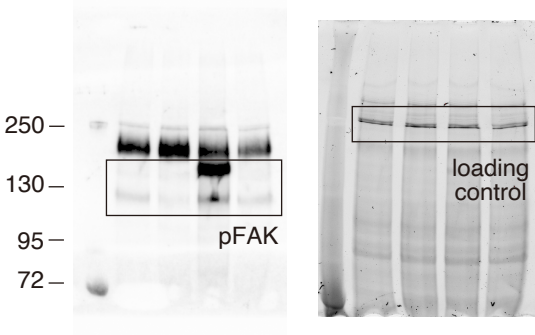

Appendix Figure 1B

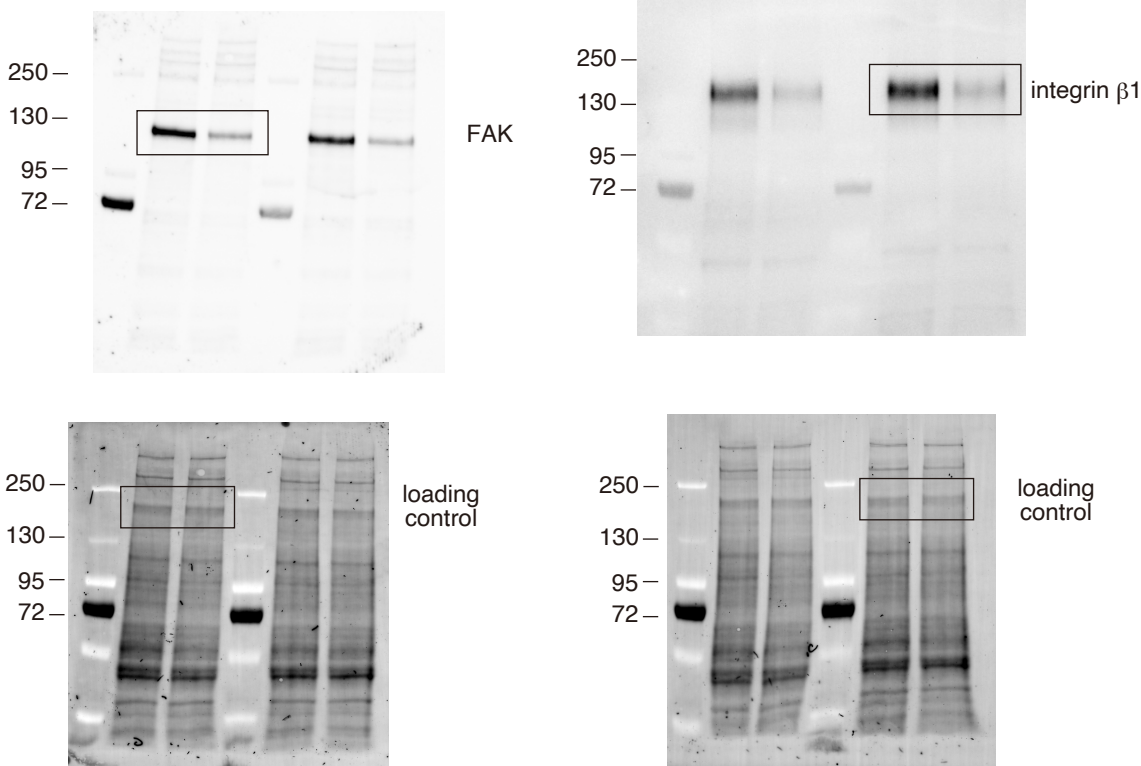

Supplement: Supplementary file 10 — Source Data for Expanded View/Appendix [file EMBJ-40-e106871-s010.zip › EMBOJ-2020-106871R2-Appendix Figure_Source_Data.pdf]
